# Assisted reproductive techniques and the risk of anorectal malformations: a German case-control study

**DOI:** 10.1186/1750-1172-7-65

**Published:** 2012-09-15

**Authors:** Nadine Zwink, Ekkehart Jenetzky, Eberhard Schmiedeke, Dominik Schmidt, Stefanie Märzheuser, Sabine Grasshoff-Derr, Stefan Holland-Cunz, Sandra Weih, Stuart Hosie, Peter Reifferscheid, Helen Ameis, Christina Kujath, Anke Rißmann, Florian Obermayr, Nicole Schwarzer, Enrika Bartels, Heiko Reutter, Hermann Brenner

**Affiliations:** 1 Division of Clinical Epidemiology and Aging Research, German Cancer Research Center, Heidelberg, Germany; 2 Department for Child and Adolescent Psychiatry, Johannes Gutenberg-University, Mainz, Germany; 3 Department of Pediatric Surgery and Urology, Centre for Child and Youth Health, Klinikum Bremen-Mitte, Bremen, Germany; 4 Department of Pediatric Surgery, Charité Universitätsmedizin Berlin, Berlin, Germany; 5 Department of Pediatric Surgery, University Hospital Würzburg, Würzburg, Germany; 6 Department of Pediatric Surgery, University of Heidelberg, Heidelberg, Germany; 7 Department of Pediatric Surgery, Klinikum Schwabing, Technical University Munich, Munich, Germany; 8 Department of Child and Adolescent Medicine, Westküstenklinikum Heide, Heide, Germany; 9 Department of Pediatric Surgery, Altonaer Kinderkrankenhaus, Hamburg, Germany; 10 Department of Pediatric Surgery, University Hospital Greifswald, Greifswald, Germany; 11 Malformation Monitoring Centre Saxony-Anhalt, Otto-von-Guericke University, Magdeburg, Germany; 12 Department of Pediatric Surgery and Urology, University Hospital for Child and Adolescent Medicine Tübingen, Tübingen, Germany; 13 SoMA e.V.; Self-help organisation for people with anorectal malformation, Munich, Germany; 14 Institute of Human Genetics, University of Bonn, Bonn, Germany; 15 Department of Neonatology, Children’s Hospital, University of Bonn, Bonn, Germany

**Keywords:** Anorectal malformation, Imperforate anus, Anal atresia, Reproductive medicine, Assisted reproductive techniques

## Abstract

**Background:**

The use of assisted reproductive techniques (ART) for treatment of infertility is increasing rapidly worldwide. However, various health effects have been reported including a higher risk of congenital malformations. Therefore, we assessed the risk of anorectal malformations (ARM) after in-vitro fertilization (IVF) and intracytoplasmic sperm injection (ICSI).

**Methods:**

Data of the German Network for Congenital Uro-REctal malformations (CURE-Net) were compared to nationwide data of the German IVF register and the Federal Statistical Office (DESTATIS). Odds ratios (95% confidence intervals) were determined to quantify associations using multivariable logistic regression accounting for potential confounding or interaction by plurality of births.

**Results:**

In total, 295 ARM patients born between 1997 and 2011 in Germany, who were recruited through participating pediatric surgeries from all over Germany and the German self-help organisation SoMA, were included. Controls were all German live-births (n = 10,069,986) born between 1997 and 2010. Overall, 30 cases (10%) and 129,982 controls (1%) were born after IVF or ICSI, which translates to an odds ratio (95% confidence interval) of 8.7 (5.9–12.6) between ART and ARM in bivariate analyses. Separate analyses showed a significantly increased risk for ARM after IVF (OR, 10.9; 95% CI, 6.2–19.0; P < 0.0001) as well as after ICSI (OR, 7.5; 95% CI, 4.6–12.2; P < 0.0001). Furthermore, separate analyses of patients with isolated ARM, ARM with associated anomalies and those with a VATER/VACTERL association showed strong associations with ART (ORs 4.9, 11.9 and 7.9, respectively). After stratification for plurality of birth, the corresponding odds ratios (95% confidence intervals) were 7.7 (4.6–12.7) for singletons and 4.9 (2.4–10.1) for multiple births.

**Conclusions:**

There is a strongly increased risk for ARM among children born after ART. Elevations of risk were seen after both IVF and ICSI. Further, separate analyses of patients with isolated ARM, ARM with associated anomalies and those with a VATER/VACTERL association showed increased risks in each group. An increased risk of ARM was also seen among both singletons and multiple births.

## Introduction

The use of assisted reproductive techniques (ART) for treatment of infertility is increasing rapidly worldwide [1,2]. Besides the use of medication for ovulation induction, ART include in-vitro fertilization (IVF) and intracytoplasmic sperm injection (ICSI). It is estimated that worldwide over 200,000 babies are annually born after ART [1-3]. While most babies born after ART are healthy, previous studies also reported on health effects, such as higher frequencies of prematurity, low birth weights and multiple births [4-8]. The risk of a malformed child after treatment is reported to be 30-40% higher than after spontaneous conception [9]. Specific associations have been reported for esophageal atresia, hypospadias (second or third degree), cleft palate, septal heart defects and the exstrophy-epispadias complex [10-12]. A few studies have also suggested an increased risk of anorectal malformations (ARM) [10,13-15]. ARM are rare birth defects concerning anus and rectum with largely unknown causes [16]. Approximately 1 in 2,500 to 1 in 3,500 new born babies are affected worldwide [17-19]. However, recently published studies did not report any information on the various types of these rare birth defects and ART. Furthermore, previous studies did not stratify for IVF and ICSI and only one accounted for plurality of birth. Also, a major problem of nearly all studies was to find a suitable population-based or register-based control group.

The aim of the present study was to assess the association between ART and ARM paying particular attention to potential variation of risk by type of ARM, and presence of associated malformations. We also aimed to clarify if and to what extent the increased risk might be explained by the impact of multiple births whose frequency is increased after ART. We addressed these questions by comparing frequencies of ART between children born with ARM and all newborn children in Germany using data from the German Network for Congenital Uro-REctal malformations (CURE-Net) [20], the population-based data from the German IVF register (DIR) [21] and the Federal Statistical Office (DESTATIS) [22]. Furthermore, we separately considered IVF and ICSI to find out possible differences in potential health effects of these procedures.

## Methods

### Study population

CURE-Net, initiated in 2009, is an ongoing, multicenter population-based study to investigate environmental and genetic risk factors, clinical implications and psychosocial outcome for congenital uro-rectal malformations. Cases with ARM are identified and recruited through participating departments of pediatric surgery all over Germany and the German self-help organisation SoMA [23]. For this analysis, we used data from patients born between 1997 and 2011 and diagnosed with an anorectal malformation. Personal interviews were conducted by a physician with parents of affected children within the first year after child-birth using a standardized questionnaire. The questionnaire contained questions about socio-demographic factors (gender of child, age of child, age of parents, country of origin, education/professional situation of the parents), family history (previous abortions, parental multiple pregnancies, consanguinity and genogram), pregnancy history and birth-related factors (gestational age, birth weight, birth position, mode of delivery, multiple pregnancy, diagnostic tests during pregnancy, treatment with ART), and lifestyle factors of the parents (smoking, alcohol consumption, illicit drug use, maternal body weight, preferred nutrition). The international classification of Krickenbeck [24] was used for standardized description of diagnostic subgroups of ARM. Associated malformations were ascertained with means of the London Dysmorphology Database [25]. Information on environmental risk factors was collected according to the core dataset of the European surveillance of congenital anomalies (EUROCAT) [26]. The study was approved by the ethics committees of the Universities of Heidelberg and Bonn. Written informed consent was obtained from the patient for publication of this report.

Data collection was initiated in 1997 by the German IVF register. This data included type and modality of performed ART procedures nationwide. Since 1998, annual submission of the data is mandatory for all German medical institutions entitled to perform ART procedures. Before 2004, data acquisition was carried out by a questionnaire. Thereafter, the software DIRpro [27] was used. Annual reports contained information about the number and type of ART procedures and multiple pregnancies, but no information on parental age, maternal body weight, or child gender.

In this study, we compared the proportion of ARM patients born after ART registered by CURE-Net with the reported nationwide proportion of live-born children following IVF or ICSI born between 1997 and 2010. According to the IVF register, 27% of all ART procedures between 1997 and 2010 were IVF and 51% were ICSI. Only a small number of parents used the combined application of IVF and ICSI (1%). Information on the exact ART type was missing in 20%. Data on the total number of live-births stratified by singletons and multiple births, were obtained from the DESTATIS database of the Federal Statistical Office of Germany [22].

### Inclusion criteria

We included patients diagnosed with ARM, including rare forms like rectal atresia/stenosis or recto-vaginal fistula. In addition, the patients were classified into ARM infants without other anomalies and ARM infants with multiple major anomalies. VATER (vertebral, anal, tracheo-esophageal, renal) and VACTERL (vertebral, anal, cardiovascular, tracheo-esophageal, renal, limb) associations were treated as a separate group.

### Statistical analysis

The t-test was used to assess possible differences in maternal age at birth between ARM cases born after ART and ARM cases born after natural conception. For calculations of other possible differences non-parametrical measurement methods were used (Mann–Whitney U-test, Fisher’s exact test). In case of a normal distribution, the mean value was calculated, otherwise the median.

We compared the frequency of ART (overall and by type of ART) between ARM patients (overall and by absence or presence of associated anomalies) and the total population of newborns in Germany. To control for potential recall and selection bias, a subgroup analysis was performed with data from multicenter and prospectively collected ARM patients born between 2009 and 2011.

Information of the IVF register on the exact ART type was missing in 20% of cases. In our analysis, the cases were allocated to the two types of ART (IVF, ICSI) according to the distribution of these types in the total data set. In addition, we conducted a sensitivity analysis to assess the impact of the unknown ART types on the risk of ARM. Moreover, separate analyses were performed for singletons and multiple births. In case of multiple births, all children were included in the analysis. Plurality of birth was taken into account due to its well-known relationship with both ART and ARM. Due to aggregated data of our control group, it was not possible to control for clustering of births within one mother. But we included a product term between ART and plurality of birth in logistic regression models to test for potential interaction between them. Analyses were performed by the statistics software SAS^©^, version 9.2 (SAS Institute Inc., Cary, N.C., USA).

## Results

In total, 295 ARM cases (172 males, 123 females) born between 1997 and 2011 were identified of whom 30 (10%) were born after ART (Table 1). Most patients had a perineal (cutaneous) fistula (36%). 45% of patients had an isolated ARM, the others had one or more associated defects. The proportion of isolated ARM was lower among ARM patients born after ART (27%) compared to those among patients born after unassisted conception (47%) (P = 0.05). 22% of patients were diagnosed with a VATER/VACTERL association. Median age (interquartile range [IQR]) of patients at the time of data acquisition was 2 (0–7) years. The majority of patients were born between the 37th and 42nd week of gestation (70%), and with a normal birth weight of 2,500 g to 3,999 g (66%). The gestational age and birth weight of ARM patients born after ART was lower compared to naturally conceived ARM patients (P = 0.002 and P = 0.01, respectively). 90% of all ARM patients were singletons and 10% were multiple births. Four of the multiple births were monozygotic twins and ten were dizygotic twins where the other twin(s) was (were) healthy. The proportion of multiple births was much higher among ARM patients born after ART (46%) than among ARM patients born after unassisted conception (6%) (P < 0.0001). Other characteristics were similar, with no significant difference between ARM patients born after ART and ARM patients born after unassisted conception.

**Table 1:** Characteristics of ARM patients born between 1997 and 2011

	**ARM patients (n = 295)**	**Unassisted conception (n = 265)**	**ART (n = 30)**	**P value**
Classification				0.27^#^
Perineal (cutaneous) fistula	107 (36%)	99 (37%)	8 (27%)	
Recto-urethral fistula	71 (24)	63 (24%)	8 (27%)	
Recto-vesical fistula	14 (5%)	10 (4%)	4 (13%)	
Vestibular fistula	37 (13%)	34 (13%)	3 (10%)	
Cloaca	17 (6%)	16 (6%)	1 (3%)	
Anal atresia without fistula	35 (12%)	29 (11%)	6 (20%)	
Anal stenosis	5 (2%)	5 (2%)	0	
Recto-vaginal fistula	3 (1%)	3 (1%)	0	
Others	6 (2%)	6 (2%)	0	
ARM differentiation				0.05^#^
Isolated malformations	132 (45%)	124 (47%)	8 (27%)	
Associated malformations	163 (55%)	141 (53%)	22 (73%)	
Vertebral anomalies	58 (20%)	48 (18%)	10 (33%)	0.06^#^
Cardiovascular anomalies	70 (24%)	64 (24%)	6 (20%)	0.82^#^
Tracheo-esophageal fistula and/or esophageal atresia	33 (11%)	27 (10%)	6 (20%)	0.12^#^
Renal (kidney) anomalies	90 (31%)	76 (29%)	14 (47%)	0.06^#^
Limb defects	28 (10%)	25 (9%)	3 (10%)	1.00^#^
VATER/VACTERL association	64 (22%)	58 (22%)	6 (20%)	1.00^#^
Gender				0.24^#^
Male	172 (58%)	151 (57%)	21 (70%)	
Female	123 (42%)	114 (43%)	9 (30%)	
Birth plurality				<0.0001^#^
Singletons	265 (90%)	249 (94%)	16 (53%)	
Twins	26 (9%)	16 (6%)	10 (33%)	
Triplets and more	4 (1%)	0	4 (13%)	
Gestational age (weeks)^*^, median (IQR)	38 (36–40)	38 (36–40)	37 (34–38)	0.002^‡^
Birth weight (gram)^*^, median (IQR)	2,990 (2,413–3,374)	3,030 (2,457–3,400)	2,702 (1,880–3,140)	0.01^‡^
Age of patient at time of study (years), median (IQR)	2 (0–7)	2 (0–6)	2 (1–8)	0.23^‡^
Maternal age (years), mean ± SD	32 ± 5.3	32 ± 5.5	32 ± 4.0	0.15^Δ^
Maternal BMI (kg/m^2^)**, median (IQR)	22.6 (20.5–25.3)	22.5 (20.6–25.2)	23.0 (20.4–26.2)	0.80^‡^

* Data was missing in one patient born after unassisted conception.

** Data was missing in two patients born after ART and ten patients born after unassisted conception.

# Calculated by the Fisher’s exact test.

‡ Calculated by the Mann–Whitney U-test.

Δ Calculated by the t-test.

ART = assisted reproductive techniques, BMI = body mass index, SD = standard deviation, IQR = interquartile range.

Information on assisted reproductive techniques in cases and controls is presented in Tables 2, 3, 4, 5, 6. According to the German IVF register, 1% of children (129,982:10,069,986) were born after IVF or ICSI (Table 2). Compared to 10% of ARM patients born after ART, this translates to a crude odds ratio for ARM after IVF or ICSI of 8.7 (95% CI 5.9–12.6) with a P value <0.0001 (Table 3). Analyses with the prospectively collected ARM patients (135 patients) confirmed our finding (OR, 8.8; 95% CI, 5.1–15.4; P < 0.0001). Separate analyses for patients with isolated ARM, ARM with associated anomalies and those with a VATER/VACTERL association all showed strong associations with ART (ORs 4.9, 11.9 and 7.9, respectively). The proportion of live-born multiple births was 10% in ARM cases (30 patients) and 3% in controls (324,630 children) (OR, 3.4; 95% CI, 2.3–5.0; P < 0.0001). This proportion was higher in patients with isolated ARM (11%) than in patients with associated anomalies (9%) or with a VATER/VACTERL association (9%). The corresponding odds ratios were 3.8, 3.0 and 3.1, respectively.

**Table 2:** Prevalence of ART in ARM cases of CURE-Net compared to all live-births in Germany

	**ARM cases in CURE-Net**			**Nationwide prevalence**		
**Year of birth**	**Total births**	**Infants born after IVF or ICSI**	**Proportion% (95% CI)**	**Total births**	**Infants born after IVF or ICSI**	**Proportion% (95% CI)**
1997	14	2	14.29 (1.78–42.81)	812,173	4,163	0.51 (0.50–0.53)
1998	8	2	25.00 (3.19–65.09)	785,034	8,406	1.07 (1.05–1.09)
1999	8	1	12.50 (0.32–52.65)	770,744	9,132	1.18 (1.16–1.21)
2000	9	0	0	766,999	9,111	1.19 (1.16–1.21)
2001	13	2	15.38 (1.92–45.45)	734,475	11,098	1.51 (1.48–1.54)
2002	13	3	23.08 (5.04–53.81)	719,250	12,303	1.71 (1.68–1.74)
2003	8	0	0	706,721	17,111	2.42 (2.39–2.46)
2004	16	2	12.50 (1.55–38.35)	705,622	8,376	1.19 (1.16–1.21)
2005	10	0	0	685,795	8,611	1.26 (1.23–1.28)
2006	19	1	5.26 (0.13–26.03)	672,724	8,958	1.33 (1.30–1.36)
2007	18	1	5.56 (0.14–27.29)	684,862	10,177	1.49 (1.46–1.51)
2008	24	2	8.33 (1.03–27.00)	682,514	9,913	1.45 (1.42–1.48)
2009	52	6	11.54 (4.35–23.44)	665,126	6,330	0.95 (0.93–0.98)
2010	45	5	11.11 (3.71–24.05)	677,947	6,293	0.93 (0.91–0.95)
2011	38	3	7.89 (1.66–21.38)	-	-	-
**Total**	**295**	**30**	**10.17 (6.97–14.20)**	**10,069,986**	**129,982**	**1.29 (1.28–1.30)**

ART = assisted reproductive techniques, IVF = in-vitro fertilization, ICSI = intracytoplasmic sperm injection, CI = confidence interval.

**Table 3:** Bivariate associations of ART and plurality with risk of ARM

**Group**		**Population-based controls N (%)**	**All ARM cases**			**Prospectively collected ARM cases in 2009 to 2011**					
			**N (%)**	**OR**_**crude**_**(95% CI)**	**P value**	**N (%)**			**OR**_**crude**_**(95% CI)**	**P value**	
ART	No	9,940,004 (99%)	265 (90%)	1.0 (Reference)	-	121 (90%)			1.0 (Reference)	-	
	Yes	129,982 (1%)	30 (10%)	8.7 (5.9–12.6)	<0.0001	14 (10%)			8.8 (5.1–15.4)	<0.0001	
Plurality of birth	Singletons	9,745,356 (97%)	265 (90%)	1.0 (Reference)	-	123 (91%)			1.0 (Reference)	-	
	Multiple births	324,630 (3%)	30 (10%)	3.4 (2.3–5.0)	<0.0001	12 (9%)			2.9 (1.6–5.3)	0.0002	
			**Isolated ARM cases**			**ARM cases with associated anomalies**			**VATER/VACTERL association**		
**Group**		**Population-based controls N (%)**	**N (%)**	**OR**_**crude**_**(95% CI)**	**P value**	**N (%)**	**OR**_**crude**_**(95% CI)**	**P value**	**N (%)**	**OR**_**crude**_**(95% CI)**	**P value**
ART	No	9,940,004 (99%)	124 (94%)	1.0 (Reference)	-	141 (87%)	1.0 (Reference)	-	58 (91%)	1.0 (Reference)	-
	Yes	129,982 (1%)	8 (6%)	4.9 (2.4–10.1)	<0.0001	22 (14%)	11.9 (7.6–18.7)	<0.0001	6 (9%)	7.9 (3.4–18.3)	<0.0001
Plurality of birth	Singletons	9,745,356 (97%)	117 (89%)	1.0 (Reference)	-	148 (91%)	1.0 (Reference)	-	58 (91%)	1.0 (Reference)	-
	Multiple births	324,630 (3%)	15 (11%)	3.8 (2.2–6.6)	<0.0001	15 (9%)	3.0 (1.8–5.2)	<0.0001	6 (9%)	3.1 (1.3–7.2)	0.005

ART = assisted reproductive techniques, OR = odds ratio, CI = confidence interval.

**Table 4:** Bivariate associations of type of ART with risk of ARM

**Group**		**Population-based controls N (%)**	**All ARM cases**			**Prospectively collected ARM cases in 2009 to 2011**		
			**N (%)**	**OR (95% CI)**	**P value**	**N (%)**	**OR (95% CI)**	**P value**
ART	No	9,940,004 (99%)	265 (90%)	1.0 (Reference)	-	121 (90%)	1.0 (Reference)	-
	IVF	44,844 (0.4%)	13 (4%)	10.9 (6.2–19.0)	<0.0001	6 (4%)	11.0 (4.8–25.0)	<0.0001
	ICSI	85,138 (0.9%)	17 (6%)	7.5 (4.6–12.2)	<0.0001	8 (6%)	7.7 (3.8–15.8)	<0.0001

ART = assisted reproductive techniques, OR = odds ratio, CI = confidence interval.

IVF = in-vitro fertilization, ICSI = intracytoplasmic sperm injection.

**Table 5:** Associations of ART with risk of ARM according to plurality of birth

**Plurality of birth**	**ART**	**Population-based controls N (%)**	**All ARM cases**			**Prospectively collected ARM cases in 2009 to 2011**		
			**N (%)**	**OR (95% CI)**	**P value**	**N (%)**	**OR (95% CI)**	**P value**
Singletons	No	9,664,317 (99%)	249 (94%)	1.0 (Reference)	-	116 (94%)	1.0 (Reference)	-
	Yes	81,039 (1%)	16 (6%)	7.7 (4.6–12.7)	<0.0001	7 (6%)	7.2 (3.4–15.4)	<0.0001
Multiple births	No	275,687 (85%)	16 (53%)	1.0 (Reference)	-	5 (42%)	1.0 (Reference)	-
	Yes	48,943 (15%)	14 (47%)	4.9 (2.4–10.1)	<0.0001	7 (58%)	7.9 (2.5–24.9)	<0.0001

ART = assisted reproductive techniques, OR = odds ratio, CI = confidence interval.

**Table 6:** Associations of type of ART with risk of ARM according to plurality of birth

**Plurality of birth**	**ART**	**Population-based controls N (%)**	**All ARM cases**			**Prospectively collected ARM cases in 2009 to 2011**		
			**N (%)**	**OR (95% CI)**	**P value**	**N (%)**	**OR (95% CI)**	**P value**
Singletons	No	9,664,317 (99%)	249 (94%)	1.0 (Reference)	-	116 (94%)	1.0 (Reference)	-
	IVF	26,743 (0.3%)	7 (3%)	10.2 (4.8–21.5)	<0.0001	3 (3%)	9.4 (3.0–29.4)	<0.0001
	ICSI	54,296 (0.6%)	9 (3%)	6.4 (3.3–12.5)	<0.0001	4 (3%)	6.1 (2.3–16.6)	<0.0001
Multiple births	No	275,687 (85%)	16 (53%)	1.0 (Reference)	-	5 (42%)	1.0 (Reference)	-
	IVF	18,060 (6%)	6 (20%)	5.7 (2.2–14.6)	<0.0001	3 (25%)	9.2 (2.2–38.3)	0.0002
	ICSI	30,883 (10%)	8 (27%)	4.5 (1.9–10.4)	0.0002	4 (33%)	7.1 (1.9–26.6)	0.0006

ART = assisted reproductive techniques, OR = odds ratio, CI = confidence interval, IVF = in-vitro fertilization, ICSI = intracytoplasmic sperm injection.

Among the ARM cases, IVF was used 13 times (4%) and ICSI 17 times (6%) (Table 4). Both procedures were used much less frequently in the reference group of all newborns in Germany (IVF 0.4% vs. ICSI 0.9%). There was a significantly increased risk for ARM after IVF (OR, 10.9; 95% CI, 6.2–19.0; P < 0.0001) as well as after ICSI (OR, 7.5; 95% CI, 4.6–12.2; P < 0.0001). Among the only prospectively collected ARM patients, corresponding ORs were 11.0 after IVF and 7.7 after ICSI.

After stratification by plurality of birth, somewhat weaker but still highly significant associations of ART with ARM were seen among both singletons (OR, 7.7; 95% CI, 4.6–12.7; P < 0.0001) and multiple births (OR, 4.9; 95% CI, 2.4–10.1; P < 0.0001) (Table 5). The same was observed with the only prospectively collected ARM patients. There was no statistically significant interaction by plurality (P = 0.70). Both types of ART were strongly and significantly related to ARM in both singletons and multiple births (Table 6).

## Discussion

To assess the association between ART and anorectal malformations, we compared data from the multicenter population-based CURE-Net with nationwide data from the German IVF register and the Federal Statistical Office. We observed a strongly and significantly increased risk of ARM after both IVF and ICSI. Further, separate analyses of patients with isolated ARM and those with associated anomalies as well as with a VATER/VACTERL association showed strongly increased risks in each group. Furthermore, a strongly increased risk of ARM was seen among both singletons and multiple births.

Our finding of an increased risk for ARM is in agreement with three previous epidemiological studies from Italy and Sweden [13-15]. All these studies observed a significantly increased risk for ARM in general (Ericson and Källén [13]: relative risk [RR], 3.1; 95% CI, 1.3–6.1; Källén et al. [14]: OR, 4.7; 95% CI, 3.2–6.9; Midrio et al. [15]: OR, 13.31; 95% CI, 4.0–39.6). However, Källén et al. [28] could not confirm their results with other cases in a second study two years later (OR, 0.90; 95% CI, 0.37–2.18), but analysis of the first and second data set together showed again a highly significant association between ART and ARM (OR, 3.11; 95% CI, 2.21–4.52; P < 0.001). Heterogeneity across both data sets was significant (χ^2^ = 43.5). One American study by Reefhuis et al. [10] stratified ARM infants by plurality of birth and reported an increased risk of ARM after ART for singletons (OR, 3.4; 95% CI, 1.2–8.3), but not for multiple births (OR, 1.3; 95% CI, 0.5–3.3). Three of the five studies mentioned above have identified their cases through the Swedish Medical Birth Register [13,14,28]. The remaining two studies obtained their information from the pediatric surgery unit of the University of Padua [15] and existing birth defects surveillance systems in the United States of America [10]. Studies were heterogeneous with respect to case numbers and control types. Case numbers ranged from 28 ARM cases [15] to 533 ARM cases [14]. Control groups were register-based [13-15,28] or from the same source populations as the case infants [10]. Control groups consisting of nationwide data were only available in the Swedish studies [13,14,28]. Period of data acquisition ranged from six years [10,15,28] to 19 years [14]. Our risk estimates were mostly higher than those of previous studies, but in the light of the overlapping confidence intervals it is unclear to what extent these differences might reflect chance variation.

The large sample size of the present study allowed differentiation between isolated ARM and ARM with associated defects which is common in approximately 64% of all ARM patients [29]. This mainly concerns anomalies of the kidney (31%), heart (24%) and vertebra (20%). To our knowledge, this study is the first study specifically addressing risk of isolated ARM, ARM with multiple anomalies and the VATER/VACTERL association. Similarly strong and significant associations were found for all three subgroups.

The previously published studies assessed the association between ARM and ART use (IVF and ICSI) in general [10], or between ARM and IVF [13-15,28]. However, to show possible differences between IVF and ICSI procedures we analysed data separately. There was a highly increased risk of ARM after both ART procedures. Normally, ICSI is used when previous IVF procedures were unsuccessful or in case of severe male infertility [30]. Although the studies by Bonduelle et al. [31] and Lie et al. [32] did not indicate an increased risk for malformed children born after ICSI compared to IVF, this procedure is often considered to be risky because it is more invasive than routine IVF [33].

Compared to other congenital malformations, ARM is not likely to be specifically associated with ART. For example, a higher risk is also reported for esophageal atresia, hypospadias (second or third degree), cleft palate, septal heart defects and the exstrophy-epispadias complex [10-12]. Our finding of an increased risk for ARM associated with ART may be due to ART per se and/or the infertility problems of couples who conceive following ART [34-37]. Regarding the inheritance in ARM families, the study by Falcone et al. [38] reported a positive family history in 1.4% of patients with ARM. In our data set, only one father and three siblings were affected with ARM, also resulting in a proportion of 1.4%. The respective four cases were all naturally conceived. In our cohort, there was no indication that families with major defects conceived via ART more often than families without major defects. However, due to missing data for our control group, it was not possible to examine clustering in more detail to confirm potential genetic pathways. Blastogenesis defects, arising in the first four weeks of pregnancy, are also conceivable as reason for the higher risk of ARM [39]. In addition, it is known that maternal overweight, obesity and diabetes, identified as risk factors for ARM in the systematic review and meta-analysis by Zwink et al. [16], occur more often in women with polycystic ovary syndrome [40-42]. This hormonal disorder is also the most common cause of infertility due to menstrual disorders.

Plurality of birth is both more common after ART and strongly related to ARM. However, our data clearly indicate that the strong relation between ART and ARM was not explained by plurality of birth. Strong associations between ART and ARM were seen among both singletons and multiple births. Compared to previous studies, our twinning rate of all ARM cases is slightly higher [43,44]. Due to lack of pertinent information in the control group, it was not possible to analyse the risk of monozygotic and dizygotic twins separately. The study by Harris et al. [44] assumed that the increased risk for twins seems to be mainly associated with monozygotic twinning.

Our study has specific strengths and limitations. Strengths include the large sample size of both ARM cases and controls. With 295 ARM cases it was possible to differentiate between ART types as well as between isolated ARM and ARM with multiple anomalies. The nationwide control group included more than 10 million births between 1997 and 2010. As data of 2011 will be only available at the end of 2012 it was not possible to include them in the analysis. Since reporting of ART procedures in Germany is mandatory by law, the registration of ART in Germany can be considered complete. To avoid any delay in reporting, we chose the updated numbers of ART published in the respective subsequent annual report. In contrast to the worldwide increase [1,2], proportions remained nearly the same over the years in Germany. However, there was a decrease in the performed procedures in 2004 because of changes in the German health system. These comprised less insurance eligibility of treatment of ART so that couples must pay half of the arising costs by themselves. In addition, services of the German health insurance company are dependent on the age of couples and available for only three treatment cycles. Different than in other countries, the embryo transfer in Germany is limited to a maximum of three embryos per treatment cycle.

Despite major efforts of nationwide recruitment of ARM cases, recruitment cannot be considered complete. In particular, active nationwide registration started in only 2009 and earlier cases could only be identified and recruited retrospectively in cooperation with hospitals and the self-help organisation SoMA. However, major selection bias from incomplete recruitment of cases could have occurred only if recruitment would have varied by application of ART for which we do not see obvious reasons. A selection bias might also have occurred in case of differences in prenatal care with potential impact on pregnancy termination between ART and natural conceptions. As all parents reported on regular prenatal control examinations an impact on the results seems unlikely. In addition, the average number of ultrasound examinations during pregnancy did not differ relevantly by history of ART in our data set (ARM patients born with ART: 15 examinations; ARM patients born without ART: 12 examinations). Nationwide data on termination of pregnancy and death of ARM patients during the first days of life are not yet available. Therefore, analyses were limited to live-births only. Possible differences in the registration of ART due to different collecting tools used in the CURE-Net register and the IVF register are also unlikely.

Retrospective collection of data could also have compromised the validity of ART information among cases. However, IVF and ICSI are very special procedures that are likely to be recalled correctly. Furthermore, a subgroup analysis with prospectively collected ARM cases born between 2009 and 2011 confirmed our findings. As we have no information about the nationwide proportion of ARM patients born with and without ART an overlap in ARM patients can not be excluded. However, since ARM is a rare malformation with an incidence of 1 in 2,500 to 1 in 3,500 live-births [17-19] an influence on the results can be neglected. Exclusion of the ARM cases from national data did not change the result.

Finally, use of controls from a nationwide registry hindered comprehensive control for potential confounding factors. We could control, however, for plurality of birth, a key risk factor of ARM, and found a strong risk increase to persist in both singletons and multiple births. It seems unlikely that this persistent strong risk could be explained by confounding. Nevertheless, our data do not allow differentiating the potential impact of ART itself or of their possible indication, such as factors associated with maternal or paternal infertility, or characteristics of the parents, such as maternal age [37,45], or of preceding treatments, such as hormonal therapies[46].

Despite its limitations, our study provides evidence for a strong association between ART and ARM. Further research should aim to elucidate the underlying mechanisms and to identify possible preventive actions to limit the frequent occurrence of ARM after application of these techniques.

## Competing interests

The authors declare that they have no competing interests.

## Authors’ contributions

Conception and design was done by EJ and HB. Acquisition of the data was done by all authors. Statistical analysis and interpretation of the data were carried out by NZ, EJ and HB. Drafting of the article was done by NZ and HB. Revision of the article was done by all authors. All authors read and approved the final manuscript.

## Funding

This work was done in the context of the “Network for Systematic Investigation of the Molecular Causes, Clinical Implications and Psychosocial Outcome of Congenital Uro-Rectal Malformations (CURE-Net)” and supported by a research grant (01GM08107) from the German Federal Ministry of Education and Research (Bundesministerium für Bildung und Forschung, BMBF): http://www.cure-net.de.
